# Supplementary material for: Global changes of miRNA expression indicates an increased reprogramming efficiency of induced mammary epithelial cells by repression of miR-222-3p in fibroblasts
Source: PeerJ. 2024 Jul 12;12:e17657. doi: 10.7717/peerj.17657 (PMC11249016; doi:10.7717/peerj.17657)
Supplement: Supplemental Information 1 — The expression or percentage of Total Raw Reads, Total Clean Reads, Low Quality Reads , Clean Reads Q20, Clean Reads Q30 in GEF and iMEC groups. [file peerj-12-17657-s001.docx]

| **Table S1 Categorization of reads of mRNAs in the different groups** | | | | | |
| --- | --- | --- | --- | --- | --- |
| Sample | Total Raw Reads | Total Clean  Reads (%) | Low Quality Reads (%) | Clean Reads Q20 (%) | Clean Reads Q30 (%) |
| GEF1 | 14, 022, 281 | 11, 046,746(78.78) | 537, 750(3.83) | 98.36 | 97.44 |
| GEF2 | 13, 297, 522 | 11, 395, 397(85.7) | 554, 968(4.17) | 98.34 | 97.41 |
| GEF3 | 12, 264,972 | 10, 131, 088(82.6) | 404, 043(3.29) | 98.68 | 97.97 |
| iMEC1 | 13, 731, 115 | 11, 793, 181(85.89) | 598, 480(4.36) | 98.12 | 97.04 |
| iMEC2 | 14, 273, 429 | 11, 967, 929(83.85) | 614, 704(4.31) | 98.21 | 97.17 |
| iMEC3 | 12, 643, 619 | 10, 746, 586(85) | 510, 993(4.04) | 98.24 | 97.23 |
