# Supplementary material for: Global changes of miRNA expression indicates an increased reprogramming efficiency of induced mammary epithelial cells by repression of miR-222-3p in fibroblasts
Source: PeerJ. 2024 Jul 12;12:e17657. doi: 10.7717/peerj.17657 (PMC11249016; doi:10.7717/peerj.17657)
Supplement: Supplemental Information 2 [file peerj-12-17657-s002.docx]

| **Table S2 Clean Reads were compared with goat reference sequence** | | | | |
| --- | --- | --- | --- | --- |
| Sample | Total Reads | Perfect Match Reads | Not Match Rate (%) | Perfect Match Rate (%) |
| GEF1 | 10, 037, 514 | 7, 870, 512 | 21.59 | 78.41 |
| GEF2 | 11, 085, 450 | 8, 225, 175 | 25.8 | 74.2 |
| GEF3 | 9, 484, 112 | 7, 320, 609 | 22.81 | 77.19 |
| iMEC1 | 11, 543, 404 | 5, 583, 749 | 51.63 | 48.37 |
| iMEC2 | 11, 319, 141 | 3, 854, 648 | 65.95 | 34.05 |
| iMEC3 | 10, 122, 908 | 5, 580, 912 | 44.87 | 55.13 |
