# Supplementary material for: Global changes of miRNA expression indicates an increased reprogramming efficiency of induced mammary epithelial cells by repression of miR-222-3p in fibroblasts
Source: PeerJ. 2024 Jul 12;12:e17657. doi: 10.7717/peerj.17657 (PMC11249016; doi:10.7717/peerj.17657)
Supplement: Supplemental Information 3 — Expression of difference mir between samples, multiplicity of difference, Cohen’s d, pval and padj values [file peerj-12-17657-s003.docx]

| **Table S3 Differentially expressed of known miRNA** | | | | | | | | | | | |
| --- | --- | --- | --- | --- | --- | --- | --- | --- | --- | --- | --- |
| miRNA | GEF1_count | GEF2_count | GEF3_count | iMEC1_count | iMEC2_count | iMEC3_count | log_2_Fold  Change | Cohen's d | pval | padj | Up/  Down |
| chi-miR-34c-3p | 1 | 1 | 0 | 55 | 24 | 47 | 6.629624499 | 3.629829761 | 4.14E-09 | 4.93E-08 | up |
| chi-miR-34b-3p | 4 | 0 | 0 | 76 | 38 | 41 | 5.975075955 | 3.349358772 | 2.87E-11 | 5.42E-10 | up |
| chi-miR-34b-5p | 6 | 10 | 6 | 204 | 143 | 119 | 5.11373702 | 4.769646106 | 1.18E-23 | 1.90E-21 | up |
| chi-miR-141 | 2088 | 2724 | 3919 | 14215 | 25032 | 73374 | 4.134931088 | 1.554028631 | 4.08E-14 | 1.46E-12 | up |
| chi-miR-34c-5p | 177 | 243 | 121 | 2560 | 1410 | 747 | 3.869077129 | 2.141239891 | 8.04E-17 | 4.30E-15 | up |
| chi-miR-10b-5p | 2728 | 6664 | 1487 | 42860 | 10411 | 27545 | 3.600899545 | 2.003382202 | 9.96E-14 | 2.89E-12 | up |
| chi-miR-200a | 4134 | 4653 | 7540 | 27525 | 16717 | 46314 | 2.910530045 | 2.31906742 | 1.74E-10 | 2.67E-09 | up |
| chi-miR-200c | 586 | 776 | 1107 | 5518 | 2597 | 5162 | 2.907682305 | 3.154161684 | 3.35E-11 | 5.97E-10 | up |
| chi-miR-190a-5p | 84 | 124 | 95 | 581 | 274 | 513 | 2.768365936 | 3.088357985 | 6.85E-15 | 3.14E-13 | up |
| chi-miR-182 | 8868 | 13028 | 15517 | 61464 | 42320 | 58777 | 2.664716463 | 5.414712265 | 3.82E-11 | 6.45E-10 | up |
| chi-miR-582-5p | 15 | 24 | 14 | 72 | 50 | 75 | 2.555058818 | 4.61169083 | 2.30E-09 | 2.95E-08 | up |
| chi-miR-429 | 3809 | 2968 | 5804 | 12240 | 12874 | 28414 | 2.550735172 | 2.081005308 | 6.54E-08 | 6.56E-07 | up |
| chi-miR-326-3p | 3 | 4 | 12 | 16 | 13 | 47 | 2.399591044 | 1.380829512 | 0.00155895 | 0.007529652 | up |
| chi-miR-181c-5p | 552 | 673 | 607 | 1798 | 1837 | 2615 | 2.383818468 | 4.480635744 | 1.58E-12 | 3.38E-11 | up |
| chi-miR-196b | 10 | 18 | 12 | 47 | 18 | 64 | 2.261850317 | 1.775566618 | 2.05E-05 | 0.00012913 | up |
| chi-miR-200b | 13616 | 20133 | 22599 | 81143 | 36750 | 53034 | 2.140939009 | 2.355353794 | 3.45E-07 | 3.08E-06 | up |
| chi-miR-10a-5p | 1729 | 2294 | 568 | 5913 | 1879 | 3780 | 2.057893253 | 1.494749409 | 1.90E-06 | 1.49E-05 | up |
| chi-miR-183 | 1302 | 1240 | 2304 | 5653 | 3724 | 4755 | 2.046525368 | 3.856545582 | 6.35E-06 | 4.43E-05 | up |
| chi-miR-708-3p | 907 | 1211 | 1556 | 3957 | 3259 | 2237 | 1.967324462 | 2.948297672 | 1.88E-05 | 0.000120598 | up |
| chi-miR-30a-3p | 163 | 272 | 194 | 637 | 403 | 500 | 1.929225924 | 3.295990182 | 4.57E-08 | 4.73E-07 | up |
| chi-miR-30a-5p | 8222 | 14083 | 11064 | 39173 | 18198 | 23905 | 1.886713057 | 2.0103433 | 1.15E-06 | 9.68E-06 | up |
| chi-miR-187 | 9 | 6 | 7 | 18 | 15 | 17 | 1.834235359 | 6.110100927 | 0.003911539 | 0.016967624 | up |
| chi-miR-99a-3p | 134 | 88 | 42 | 202 | 143 | 226 | 1.817910962 | 2.305502711 | 1.27E-06 | 1.02E-05 | up |
| chi-miR-628-5p | 108 | 129 | 149 | 348 | 219 | 358 | 1.815638897 | 3.168505819 | 2.05E-06 | 1.57E-05 | up |
| chi-miR-30f-5p | 67 | 100 | 146 | 327 | 140 | 169 | 1.535534938 | 1.407477556 | 0.002293219 | 0.010223935 | up |
| chi-miR-1839 | 736 | 731 | 763 | 1181 | 1244 | 1844 | 1.534236258 | 2.623635087 | 1.13E-05 | 7.39E-05 | up |
| chi-miR-378-3p | 16852 | 21328 | 12215 | 38090 | 21132 | 31121 | 1.504277432 | 1.948386299 | 1.72E-07 | 1.58E-06 | up |
| chi-miR-196a | 20 | 27 | 25 | 50 | 16 | 77 | 1.500428876 | 1.087428428 | 0.004525498 | 0.019114273 | up |
| chi-miR-378-5p | 80 | 61 | 50 | 119 | 88 | 130 | 1.466447207 | 2.592714728 | 3.07E-05 | 0.000189761 | up |
| chi-let-7c-5p | 5468 | 5607 | 1711 | 6367 | 5540 | 7777 | 1.383806513 | 1.309646266 | 9.37E-05 | 0.000547055 | up |
| chi-miR-504 | 272 | 282 | 231 | 379 | 311 | 443 | 1.162903651 | 2.299924938 | 0.000139437 | 0.000785249 | up |
| chi-miR-181d | 194 | 309 | 186 | 391 | 225 | 337 | 1.11455146 | 1.140567022 | 0.000664424 | 0.00344 | up |
| chi-let-7b-5p | 38565 | 47648 | 27554 | 55527 | 38312 | 51515 | 1.029474163 | 1.102586926 | 0.000142686 | 0.000789695 | up |
| chi-miR-29a-3p | 63515 | 57974 | 36904 | 14182 | 13269 | 18297 | -1.10936296 | 3.715046964 | 7.13E-05 | 0.000423834 | down |
| chi-miR-188-5p | 1203 | 983 | 493 | 279 | 139 | 343 | -1.163223247 | 2.391093726 | 0.000619456 | 0.003259761 | down |
| chi-miR-502b-3p | 125 | 156 | 48 | 37 | 23 | 25 | -1.196953211 | 2.049515808 | 0.006618731 | 0.025909911 | down |
| chi-miR-128-5p | 204 | 275 | 116 | 48 | 41 | 52 | -1.358296479 | 2.680390979 | 0.000121635 | 0.00069723 | down |
| chi-miR-145-3p | 561 | 326 | 393 | 116 | 109 | 84 | -1.38337497 | 3.74496156 | 0.000891938 | 0.004473627 | down |
| chi-miR-3432-5p | 2982 | 2676 | 1987 | 832 | 394 | 634 | -1.409375856 | 4.915138465 | 8.42E-06 | 5.63E-05 | down |
| chi-miR-30c-3p | 198 | 205 | 73 | 61 | 23 | 16 | -1.497335075 | 2.26893348 | 0.004018376 | 0.01719865 | down |
| chi-miR-455-5p | 6506 | 4421 | 3315 | 927 | 1035 | 1077 | -1.518855488 | 3.255578561 | 8.38E-06 | 5.63E-05 | down |
| chi-miR-143-5p | 39 | 38 | 26 | 9 | 7 | 6 | -1.54591809 | 5.164371376 | 0.007226274 | 0.027614689 | down |
| chi-miR-143-3p | 160520 | 96758 | 144400 | 21485 | 32342 | 32315 | -1.592847812 | 4.408553628 | 0.000185416 | 0.001008787 | down |
| chi-miR-3432-3p | 312 | 310 | 145 | 64 | 37 | 50 | -1.640762752 | 3.000098831 | 3.90E-06 | 2.78E-05 | down |
| chi-miR-27b-5p | 4406 | 4525 | 1603 | 696 | 398 | 918 | -1.697997117 | 2.399549082 | 7.31E-07 | 6.34E-06 | down |
| chi-miR-29a-5p | 51 | 190 | 57 | 36 | 8 | 12 | -1.705993512 | 1.425590362 | 0.005337481 | 0.021965788 | down |
| chi-miR-16a-3p | 90 | 77 | 37 | 9 | 12 | 17 | -1.715097154 | 2.803110415 | 0.000471133 | 0.002520562 | down |
| chi-miR-147-5p | 32 | 24 | 45 | 3 | 4 | 15 | -1.741263237 | 2.97531213 | 0.013897314 | 0.049022393 | down |
| chi-miR-25-5p | 539 | 759 | 290 | 119 | 55 | 105 | -1.819186507 | 2.603124002 | 1.63E-07 | 1.53E-06 | down |
| chi-miR-130b-5p | 2013 | 2468 | 911 | 458 | 203 | 246 | -1.843800082 | 2.603284145 | 1.22E-06 | 1.01E-05 | down |
| chi-miR-3958-3p | 29 | 37 | 28 | 5 | 1 | 12 | -1.844963707 | 4.816304304 | 0.006502281 | 0.025768297 | down |
| chi-let-7e-3p | 105 | 96 | 50 | 12 | 14 | 16 | -1.868038711 | 3.33197329 | 3.94E-05 | 0.00023863 | down |
| chi-miR-92a-5p | 1198 | 2556 | 884 | 333 | 118 | 340 | -1.895684288 | 2.020424536 | 2.15E-06 | 1.60E-05 | down |
| chi-miR-140-3p | 16510 | 14729 | 11967 | 2827 | 2082 | 2266 | -1.92775312 | 7.3156877 | 5.68E-10 | 7.93E-09 | down |
| chi-miR-26b-3p | 316 | 423 | 219 | 61 | 31 | 60 | -2.000775865 | 3.672677422 | 2.86E-09 | 3.54E-08 | down |
| chi-miR-455-3p | 1486 | 1112 | 739 | 170 | 149 | 171 | -2.067939298 | 3.591289157 | 1.30E-10 | 2.09E-09 | down |
| chi-miR-454-5p | 203 | 296 | 78 | 38 | 10 | 28 | -2.219313286 | 2.141054968 | 3.55E-06 | 2.59E-05 | down |
| chi-miR-671-3p | 301 | 304 | 140 | 41 | 25 | 30 | -2.243237001 | 3.248209192 | 2.11E-09 | 2.82E-08 | down |
| chi-miR-193a | 4054 | 2520 | 4600 | 460 | 461 | 562 | -2.356356849 | 4.230019473 | 3.37E-08 | 3.61E-07 | down |
| chi-miR-193b-3p | 4058 | 2528 | 4609 | 462 | 461 | 563 | -2.356448891 | 4.23866676 | 3.28E-08 | 3.61E-07 | down |
| chi-miR-100-5p | 10985 | 8995 | 5250 | 886 | 968 | 1024 | -2.401373606 | 3.617501488 | 1.08E-13 | 2.89E-12 | down |
| chi-let-7b-3p | 597 | 525 | 318 | 71 | 48 | 45 | -2.428088868 | 4.133022505 | 1.46E-11 | 2.92E-10 | down |
| chi-miR-1296 | 295 | 295 | 201 | 52 | 12 | 15 | -2.686156522 | 5.721229034 | 7.08E-08 | 6.89E-07 | down |
| chi-miR-222-3p | 67773 | 139972 | 25841 | 9094 | 4176 | 6596 | -2.782162655 | 1.743571822 | 2.56E-10 | 3.73E-09 | down |
| chi-miR-221-5p | 1192 | 4366 | 846 | 256 | 123 | 140 | -2.832785922 | 1.428925572 | 1.57E-08 | 1.80E-07 | down |
| chi-miR-27a-5p | 2354 | 3079 | 1697 | 210 | 105 | 298 | -2.929576803 | 4.401357401 | 2.32E-19 | 2.49E-17 | down |
| chi-miR-2335 | 26 | 20 | 13 | 3 | 0 | 2 | -2.932606106 | 3.808866326 | 0.001720317 | 0.008003215 | down |
| chi-miR-199a-5p | 2920 | 3601 | 1035 | 159 | 121 | 324 | -2.948738084 | 2.457378731 | 7.23E-14 | 2.32E-12 | down |
| chi-let-7c-3p | 30 | 25 | 9 | 1 | 1 | 2 | -3.285793469 | 2.574846453 | 0.000835898 | 0.004259097 | down |
| chi-miR-191-3p | 342 | 301 | 151 | 26 | 11 | 13 | -3.288981999 | 3.476673472 | 3.07E-14 | 1.23E-12 | down |
| chi-miR-155-5p | 13275 | 27114 | 4009 | 1101 | 587 | 732 | -3.383710547 | 1.701412564 | 8.63E-13 | 1.98E-11 | down |
| chi-miR-214-3p | 1632 | 1262 | 480 | 53 | 58 | 66 | -3.485399004 | 2.56224536 | 1.07E-18 | 6.85E-17 | down |
| chi-miR-145-5p | 7 | 8 | 8 | 1 | 0 | 0 | -3.798289838 | 12.70170592 | 0.013658611 | 0.048715711 | down |
| chi-miR-214-5p | 299 | 217 | 83 | 5 | 8 | 11 | -3.899530683 | 2.484954963 | 1.31E-13 | 3.24E-12 | down |
| chi-miR-487b-3p | 2 | 19 | 11 | 0 | 0 | 1 | -4.250667996 | 1.714303917 | 0.006767882 | 0.02617458 | down |
| chi-miR-665 | 9 | 4 | 4 | 0 | 0 | 0 | -4.272541606 | 2.776088375 | 0.013042525 | 0.047041016 | down |
| chi-miR-155-3p | 10 | 8 | 2 | 0 | 0 | 0 | -4.405036621 | 2.264554068 | 0.009709901 | 0.036669155 | down |
| chi-miR-296-3p | 4529 | 5152 | 2321 | 151 | 77 | 77 | -4.57546798 | 3.705094109 | 2.22E-35 | 7.14E-33 | down |
| chi-miR-100-3p | 491 | 418 | 113 | 9 | 6 | 8 | -4.710372938 | 2.348547754 | 9.04E-19 | 6.85E-17 | down |
