# Supplementary material for: Global changes of miRNA expression indicates an increased reprogramming efficiency of induced mammary epithelial cells by repression of miR-222-3p in fibroblasts
Source: PeerJ. 2024 Jul 12;12:e17657. doi: 10.7717/peerj.17657 (PMC11249016; doi:10.7717/peerj.17657)
Supplement: Supplemental Information 4 — Differential expression of down-regulated miRNA enriched KEGG pathway. [file peerj-12-17657-s004.docx]

| **Table S4 The KEGG analysis results of differential downregulated miRNAs** | | |
| --- | --- | --- |
| Term Name | P-value | FDR |
| Apoptosis | 7.59E-08 | 1.04E-06 |
| Insulin signaling pathway | 9.51E-07 | 1.01E-05 |
| GnRH signaling pathway | 0.000169685 | 0.000781928 |
| Progesterone-mediated oocyte maturation | 0.000435666 | 0.001834101 |
| Oxytocin signaling pathway | 0.00068407 | 0.002591864 |
| Prolactin signaling pathway | 0.001421299 | 0.004945539 |
| Biosynthesis of unsaturated fatty acids | 0.001447551 | 0.00498601 |
| Wnt signaling pathway | 0.002212794 | 0.007186311 |
| JAK-STAT signaling pathway | 0.012345863 | 0.032595257 |
| cAMP signaling pathway | 0.014242188 | 0.036243181 |
