# Supplementary material for: Global changes of miRNA expression indicates an increased reprogramming efficiency of induced mammary epithelial cells by repression of miR-222-3p in fibroblasts
Source: PeerJ. 2024 Jul 12;12:e17657. doi: 10.7717/peerj.17657 (PMC11249016; doi:10.7717/peerj.17657)
Supplement: Supplemental Information 5 — Differential expression of -regulated miRNA enriched KEGG pathway. [file peerj-12-17657-s005.docx]

| **Table S5 The KEGG analysis results of differential upregulated miRNAs** | | |
| --- | --- | --- |
| Term Name | P-value | FDR |
| Cell cycle | 5.90E-17 | 5.03E-15 |
| Focal adhesion | 3.67E-11 | 1.16E-09 |
| Adherens junction | 8.94E-11 | 2.18E-09 |
| MAPK signaling pathway | 8.93E-10 | 1.32E-08 |
| Autophagy - animal | 2.58E-09 | 3.25E-08 |
| AMPK signaling pathway | 2.20E-08 | 2.50E-07 |
| PI3K-Akt signaling pathway | 8.23E-08 | 8.25E-07 |
| IL-17 signaling pathway | 9.19E-06 | 5.60E-05 |
| Insulin resistance | 6.39E-05 | 0.000311318 |
| TGF-beta signaling pathway | 0.00017594 | 0.000759435 |
